# Construction of an odds model of coronary heart disease using published information: the Cardiovascular Health Improvement Model (CHIME)

**DOI:** 10.1186/1472-6947-8-49

**Published:** 2008-10-31

**Authors:** Christopher J Martin, Paul Taylor, Henry WW Potts

**Affiliations:** 1 Centre for Health Informatics and Multiprofessional Education, University College London, Archway Campus, Highgate Hill, London, N19 5LW, UK

## Abstract

**Background:**

There is a need for a new cardiovascular disease model that includes a wider range of relevant risk factors, in particular lifestyle factors, to aid targeting of interventions and improve population models of the impact of cardiovascular disease and preventive strategies. The model needs to be applicable to a wider population including different ethnic groups, different countries and to those with and without cardiovascular disease. This paper describes the construction of the Cardiovascular Health Improvement Model that aims to meet these requirements.

**Method:**

An odds model is used. Information was taken from 2003 mortality statistics for England and Wales, the Health Survey for England 2003 and published data on relative risk in those with and without CVD and mean blood pressure values in hypertensives. The odds ratios used were taken from the INTERHEART study.

**Results:**

A worked example is given calculating the 10-year coronary heart disease risk for a 57 year-old non-diabetic male with no personal or family history of cardiovascular disease, who smokes 30 cigarettes a day and has a systolic blood pressure of 137 mmHg, a total cholesterol (TC) of 6.2 mmol/l, a high density lipoprotein (HDL) of 1.3 mol/l, and a body mass index of 21. He neither drinks regularly nor exercises. He can give no reliable information about his mental health or fruit and vegetable intake. His 10-year risk of CHD death is 2.47%.

**Conclusion:**

This paper demonstrates a method for developing a CHD risk model. Further improvements could be made to the model with additional information. The method is applicable to other causes of death.

## 1 Background

There are several reasons for calculating the risk of cardiovascular disease in an individual or a population. Health care providers need to model future patterns of need for health services, and to identify the cost effectiveness of different intervention strategies.[1,2] Insurance companies and pension funds must evaluate risk in both individuals and populations when assessing portfolio risks. In clinical medicine, cardiovascular risk is increasingly accepted as the appropriate criterion to use to identify those who will most benefit from interventions designed to prevent cardiovascular disease and death.[3,4] Another, perhaps overlooked requirement, is to inform shared decision-making with patients.[5]

This paper describes a cardiovascular disease (CVD) model which has been developed specifically for use in consultations with patients as an aid to risk communication and to shared decision making. Most CVD models focus on coronary heart disease (CHD) events, such as myocardial infarction. However, it is sometimes difficult to categorize an individual as either having or not having experienced a CHD event, since the collection of data on such events varies according to methods and definitions used. Consequently, an evaluation of all CHD or CVD events will be less reliable than one with a more concrete outcome measure such as CHD and CVD death.[6] The model we propose therefore estimates death from CHD.

There are a variety of CVD risk estimators available, the best known are summarized in Table 1. Each has strengths and weaknesses. [6-12] The principal problems include limited applicability to different geographic areas or ethnic groups, application to men but not women, and the omission of important risk factors.[13,14]

**Table 1:** Major cardiovascular risk models and their inputs and outputs.

**Risk equation**	**Risk factors included**	**Risks evaluated**
**Framingham (Anderson)**[7]	Age, gender, smoking, blood pressure (BP), total cholesterol (TC)/high density lipoprotein (HDL) ratio, diabetes, left ventricular hypertrophy (LVH)	4 to 12-year risk of CHD events and death, all CVD events and death, all cerebrovascular events, myocardial infarction events.

**Framingham (D'Agostino)**[12]	Age, gender, smoking, BP, TC/HDL ratio, alcohol, existing CVD, menopausal status (women), triglycerides in women	2-year risk of CHD events.

**Whitehall equation**[32]	Age, gender, TC, BP, cigarettes per day	5 or 10-year risk of CHD event.

**Systematic Coronary Risk Evaluation (SCORE)**[6]	Age, gender, smoking, BP, TC and residence in a 'high' or 'low' risk country.	10-year risk of death from CHD or CVD.

**Munster Heart Study (PROCAM)**[33]	Age, smoking, BP, low density lipoprotein (LDL), HDL, triglycerides, gamma glutamyl transferase (γGT), diabetes, existing angina, family history	Major coronary event.

**Ethrisk**[11]	Age, gender, smoking, BP, TC/HDL ratio, diabetes, LVH and ethnic group	10-year risk of CHD event.

**ASSIGN**[10]	Age, gender, cigarettes per day, systolic BP, TC/HDL ratio, family history, SIMDSC10 deprivation score	10-year risk of CVD.

**QRisk**[9]	Age, gender, smoking, BP, TC/HDL, body mass index (BMI), family history, treatment with antihypertensive drugs, Townsend area deprivation score.	10-year risk of CVD events.

The best known estimators are the Framingham equations. These have been criticized for their inaccuracy in some countries, in particular Southern Europe where they tend to over-estimate risk significantly.[14] This variation is an inevitable consequence of the exclusion of significant risk factors from the model. If a model is derived in a particular population, the prevalence and impact of any missing risk factors is tacitly embedded in coefficients of the risk equations. When applied to a population with different prevalences or one in which risk factors have different impacts, the model's predictions will be less accurate. Attempts have been made to recalibrate the Framingham equations for different ethnic groups in the United States and the United Kingdom. [11,15] However, the recalibrated equations have not been validated and questions about their applicability to other geographic areas remain unanswered.

The models in Table 1 all include age, gender, blood pressure, cholesterol, cigarette consumption and diabetes as risk factors. All omit some important independent risk factors such as family history, existing CVD, obesity but also diet, alcohol consumption and exercise. We are particularly interested in risk factors related to lifestyle: if an estimate of risk is to be used in consultations as part of discussions with patients about lifestyle modification, it is important that the estimate should include the fullest possible range of risk factors relating to lifestyle.

To improve CVD risk equations, it is necessary both to expand the number of risk factors used and to devise a method of calibrating the results to different populations. Including additional risk factors should improve the accuracy at the level of the individual and increase the portability of any risk equation to different populations, however, there will always be some residual variability not accounted for by included risk factors. National mortality statistics can be regarded as containing all possible information about risk, both known and unknown. Recalibrating such national mortality statistics according to the mean values for a broad set of known risk factors will leave a residual value for the remaining variability due to unknown factors. The 2003 Health Survey for England collected information on cardiovascular disease risk factors and prevalence which can be used to recalibrate national mortality statistics in this way.[16,17]

This paper describes how to take publicly available information on CHD prevalence, CHD death rates and CHD risk factors, and use it to calculate the risk of coronary heart disease for individuals, using an approach that should be applicable in different geographical areas and different ethnic groups.

## 2 Method

In this section, we first explain the mathematics underlying our approach and then describe how the data items required by the model were obtained. The approach uses an odds model. The odds of dying of cardiovascular disease in time *t *are:

$$O_{t}=\frac{P_{t}}{1-P_{t}}$$

where P_t _is the probability of dying of cardiovascular disease in time *t*.

If we know the average odds of death in time *t *for the given population (Pop*O*_*t*_), we can calculate an odds ratio adjustment for any individual based on known risk factors, and use it to estimate the odds for the individual as:

*IndO*_*t *_= *PopO*_*t*_.*IndOR*

The odds ratio for the individual (Ind*OR*) is the product of the odds ratios for each of *n *risk factors:

$$IndOR=\prod_{i=1}^{n}OR_{i}$$

This is often expressed as:

*IndOR *= *e*^*λ*^

where λ is the sum of terms corresponding to each risk factor, each term consisting of a coefficient *β *– a measure of the contribution of the risk factor – adjusted according to the extent to which the risk factor is present in an individual compared to the average for the population in question. Thus:

$$\lambda =\sum_{i=1}^{i=n}\beta_{i}.(s_{i}-\bar{s_{i}})$$

where *β*_*i *_is the coefficient associated with the *i*th risk factor (equal to the log of the odds ratio), *s*_*i *_is the value for the individual of the risk factor and ${\bar{s}}_{i}$ is the average population level. This is a well established method of adjusting models to different populations, used in SCORE and ETHRISK.[11,18,19]

In logistic regression, *β*_*i *_are constants representing a linear relationship between the log odds and the level of the risk factor. This approach can be applied whether the risk factor, *s*_*i*_, is a continuous, categorical or a binary variable. However, in the literature, continuous risk factors are frequently treated as categorical variables: for example, a study might give odds ratios for each quintile of waist-to-hip ratio (using the first quintile as the reference category). While these values could be used directly, that would produce artefacts in the model near quintile boundaries, so it is sensible to convert back to a continuous variable by applying smoothing. However, with some risk factors, the resultant relationship is not linear. Our approach here is to calculate an interpolated and smoothed function for how the odds ratio varies with *s*_*i *_(which is equivalent to considering *β*_*i *_not as a constant but as a function of *s*_*i*_). In these cases, instead of calculating a term

(1) $$\xi_{i}=\beta_{i}.(s_{i}-\bar{s_{i}})$$

we calculate a term:

(2) $$\xi_{i}=\ln\left(\frac{IndOR}{PopOR}\right)$$

which is the log of the odds ratio for the individual for the *i*th risk factor (i.e. associated with the measured value *s*_*i*_), divided by the odds ratio for the mean level in the population of the same risk factor (i.e. associated with ${\bar{s}}_{i}$). These terms are referred to as the log of the normalized odds ratio (LNOR) and are represented by *ξ*_*i*_. So our λ is calculated as:

(3) $$\lambda =\sum_{i=1}^{i=n}\xi_{i}$$

The model therefore requires:

• estimates of the baseline mortality from CHD (*PopO*_*t*_);

• a set of risk factors with known odds ratios;

• LNORs for each risk factor.

In the following three subsections of the paper we explain (1) how estimates were derived for baseline CHD mortality, (2) choice of a set of risk factors, and (3) how adjusted LNORs were determined for each risk factor. We then go through a worked example for an individual patient.

### 2.1 Baseline Mortality

The mortality of CHD was extracted from the UK national mortality statistics 2003.[20] The ICD-10 codes included for CHD were I20–I25 inclusive. A probability of death from CHD for each age band was calculated for each gender by dividing the number of deaths in the age band by the number of individuals in the population in that age band. The annual death rates for each age from 35 years-old upwards were then smoothly interpolated using methods described below. The probability of death was set to zero below the age of 35 as the death rates in this group were negligible.

National mortality statistics include all CHD deaths in the population. This includes CHD death in those with pre-existing CVD as well as those who were free of CVD. If we know the proportion of the population who had pre-existing CVD, the number of CHD deaths and the relative risk of CHD in those with CVD as compared to those without, then separate estimates can be made of the baseline CHD mortality in the two groups. If:

M = Mortality from CHD for that age and gender.

M_1 _= Mortality from CHD in individuals without prior CVD.

M_2 _= Mortality from CHD in individuals with prior CVD.

Pr = Prevalence of CVD for that age and gender.

and RR = the relative risk of CHD in those with a prior history of CVD compared to those without.

Then:

M = M_1_.(1 - Pr) + M_2_.Pr

Since

M_2 _= M_1_.RR

we have

M = M_1_.(1 - Pr) + M_1_.RR.Pr

Thus:

(4) $$M_{1}=\frac{M}{\left(1-\mathrm{Pr}+RR.\mathrm{Pr}\right)}$$

We calculate baseline estimates of CHD mortality for an individual with given age, gender and CVD status. M is then calculated from national mortality statistics and PR from the Health Survey for England 2003, interpolated using the approach described below. A figure of 3.3 was used for the RR for CHD death or sudden death in those with existing CHD, taken from the Framingham study. [21]

#### 2.1.1 Smooth interpolation of mortality and prevalence rates

The prevalence rates for CVD are given in the Health Survey for England 2003 in 10-year age bands. The mortality statistics are given in 5-year age bands. To obtain accurate annual estimates of baseline mortality rates it is necessary to interpolate from these totals. A number of different methods were explored, including simple linear, cubic spline and fractional polynomials, but all proved unsatisfactory.[22,23]

Linear interpolation using the mid points of the 5-year age bands fails to preserve the area under the curve within the age bands where there is a high rate of change of risk. Also, the effect of the sharp changes in risk at the the mid-point inflections is magnified in subsequent calculations to give artefactual 'edge effects'.

Interpolating with a spline function would generate a polynomial for each age band, requiring thirty or forty coefficients to describe a mortality curve from age fifteen to ninety. In addition, ensuring that the average value matches the average value for the age band, can result in values below zero at very low risks. Fractional polynomials can be fitted for narrow intervals, but as the polynomial functions may tend towards plus or minus infinity, it is difficult to fit one fractional polynomial over the wide age ranges needed without experiencing what is called Runge's phenomenon, where the included data points are fitted very well, but with dramatic error between them. [24]

A key problem is that the area under the cumulative mortality curve needs to be conserved. A two-step process was developed in which a smoothing algorithm generates a curve which is then modeled as a weighted sum of sixteen Normal distribution curves.

An interpolated curve is first generated by redistributing the area under the stepped curve obtained from the initial data: the sharpest angle in each age band is identified, by finding the biggest change in angle and dividing it by the absolute value of y-axis point. That data point is shifted towards the further of the two adjacent data points. The amount by which the data point is increased or decreased is then redistributed to the other data points in the age band. The process is repeated, iteratively reducing the maximum angle and resulting in a smooth curve that does not fall below zero, and preserves the area under the curve in each age band.

**Figure 1:**
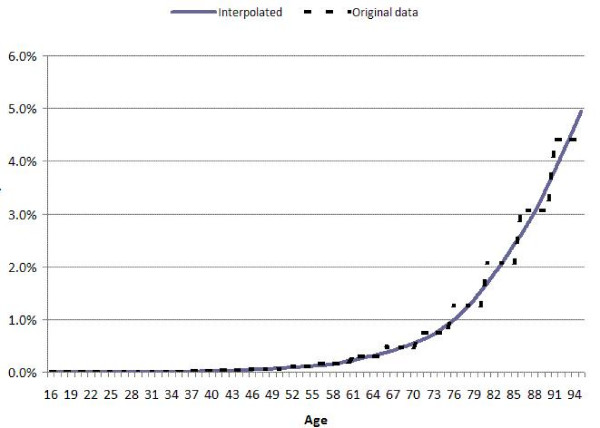
showing the resulting curve generated for the risk of death from coronary heart disease in men compared to the original stepwise 5-year age band mortality.

Below the age of 35, the prevalence and mortality are set to zero. For ages 35 and above, a function of a set of normal distribution curves was generated from the points in the smoothed curve. This produces a more tractable equation. Generating all data points prior to finding the best fit function prevents Runge's phenomenon. The parameters and weightings – determined by the least squares method – of the Normal distributions are shown in Table 2. Figure 1 shows the result of the interpolation of coronary heart disease results against the original stepwise mortality for the five-year age bands. This curve can be generated using no more than 16 numbers, does not violate the normal bounds of probability, is not affected by Runge;s phenomenon, and preserves the total risk in each age band.

**Table 2:** The coefficients for the normal distribution curves for mortality and prevalence functions.

**Normal distribution** **(μ, δ)**	**Male CVD Prevalence**	**Female CVD Prevalence**	**Male CHD mortality**	**Female CHD mortality**
**Constant**	-4.46924	-3.7276	0.028432	-0.06621

**(20,5)**	-1.66148	-1.40524	0.027838	-0.07657

**(30,5)**	2.566571	2.165315	0.02106	0.003326

**(40,5)**	3.595888	3.149946	0.020598	0.007303

**(50,5)**	4.457566	3.667629	0.019311	0.000279

**(60,5)**	6.476155	5.555759	0.011118	-0.00443

**(70,5)**	11.12182	8.868936	0.021131	-0.00807

**(80,5)**	10.75465	9.161873	0.015343	-0.00909

**(90,5)**	-2.37347	-2.19787	-0.03721	-0.03203

**(25,10)**	-37.9802	-31.3032	0.223831	-0.74789

**(45,10)**	4.541968	6.42029	0.193671	-0.03395

**(65,10)**	10.22757	12.67255	0.026298	-0.18066

**(85,10)**	21.14716	23.89516	0.160345	-0.36785

**(20,15)**	204.0157	170.2051	-1.38416	3.377389

**(50,15)**	42.36992	30.87008	-1.85748	0.545179

**(80,15)**	-91.5426	-90.6798	-2.89911	-0.95328

**(90,30)**	429.1505	365.718	5.065048	8.756594

### 2.2 Risk factors

The most comprehensive data on the odds ratios associated with a set of risk factors has come from the INTERHEART study, which collected data from 15,152 patients admitted for a first MI at 262 centres in 52 countries across the world, and 14,820 matched controls. [25] The INTERHEART study identified nine risk factors in addition to age and gender, which accounted for 90% of population attributable risk (PAR) in men and 94% in women for first myocardial infarction. We assume that the odds ratios for the risk of MI will be very similar to the odds ratios for CHD in general. The nine risk factors identified in addition to age and gender were: smoking status, a diagnosis of hypertension, apolipoprotein B/apolipoprotein A1 ratio, diabetes mellitus, waist/hip circumference ratio, alcohol consumption, consumption of fresh fruit and vegetables, exercise and psychosocial stress. A tenth factor, a family history of CHD, is also given in the paper but was omitted from the list of nine as it had minimal impact on the PAR. It has been included here as it enhances the individualization of the calculation regardless of the overall impact on the calculated population mortality.

The nine risk factors identified in the INTERHEART study are shown in Table 3 along with the unadjusted odds ratios.

**Table 3:** The risk factors identified in the INTERHEART study, their definitions and odds ratios.

**Risk factor**	**Definition**	**OR**
Smoking status[25]	Current versus non-smoker	2.87
ApoB/ApoA1 ratio (personal communication)		
2nd versus 1^st ^quintile	<0.57	1.42
3rd versus 1^st ^quintile	0.57–0.69	1.84
4th versus 1^st ^quintile	0.69–0.82	2.41
5th versus 1^st ^quintile	0.98	3.25
History of hypertension[25]	Self reported	1.91
Diabetes mellitus[25]	Self reported	2.37
Abdominal obesity[29]	Waist to hip ratio	
2nd versus 1^st ^quintile	Women: 0.79–0.84, men: 0.87–0.91	1.03
3rd versus 1^st ^quintile	Women: 0.84–0.89, men: 0.91–0.94	1.083
4th versus 1^st ^quintile	Women: 0.89–0.94, men: 0.94–0.98	1.379
5th versus 1^st ^quintile	Women: 0.94->, men: 0.98->	1.666
Psychosocial factors[25]	An unreported algorithm	2.67
Family history of premature CVD	A first degree relative <55 for men and <60 for women	1.45
Consumption of fruit and vegetables[25]	Daily consumption versus not	0.70
Regular alcohol consumption[25]	At least three days a week	0.91
Regular physical activity[25]	At least four hours a week	0.86

### 2.3 Estimating the adjustments for each risk factor

We use an odds model in which the impact of risk factors on an individual's risk for CHD death is determined as the product of a set of coefficients, one for each risk factor. The coefficients (the log of the normalized odds ratios, LNOR) provide a measure of the influence of the measured risk factor for that individual.

The population mean values were derived from the Health Survey for England 2003 for different gender and age groups, with the values interpolated using polynomials; details are given in tables 4. The following subsections detail the calculations of the LNORs for each of the risk factors used.

#### 2.3.1 ApoB/ApoA1 ratio

Total cholesterol (TC) and high density lipoprotein (HDL) values are the most common measures of lipid level used in calculating CVD risk. However, the INTERHEART study found that using the ratio of apolipoprotein B (ApoB) and apolipoprotein A1 (ApoA1) is a more sensitive measure of risk than the TC/HDL ratio.

**Figure 2:**
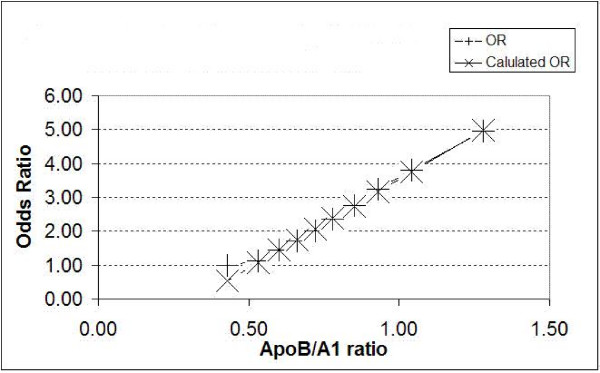
showing the relationship between the odds ratio for first MI and deciles of ApoB/A1 ratio with respect to the first decile.

The INTERHEART study explored the relationship between the deciles of ApoB/A1 ratio and the odds ratio for MI compared to the first decile. The relationship is plotted on a doubling scale in the original paper, but it would appear that the relationship between the odds ratio and the ApoB/A1 ratio is linear from the second decile upwards, but with the odds ratio having a floor of 1 from just below the second decile. This can be seen in Figure 2.

Linear regression gives the equation:

(5) $$OR=\{\begin{array}{cc} -1.7+5.23.x & if\;x>0.5\\ 1 & if\;x\leq 0.5 \end{array}$$

where *x *is the ApoB/ApoA1 ratio.

The LNOR *ξ*_*i *_for ApoB/A1 ratio is the log of the of the normalized odds ratio for an individual's ApoB/A1 ratio (IndOR_Apo_) divided by the odds ratio for the population average (PopOR_Apo_) calculated from the above equation. From equation (2), LNOR *ξ*_*Apo *_coefficient is then:

$$\xi_{Apo}=\ln\left(\frac{IndOR_{Apo}}{PopOR_{Apo}}\right)$$

The ApoB/A1 ratio is often not known as it is more customary to use TC/HDL clinically. An approximate conversion factor is applied: [26]

(6) $$\text{ApoB/A1 ratio }=\frac{\text{(TC/HDL})}{4.41}$$

#### 2.3.2 Smoking

**Figure 3:**
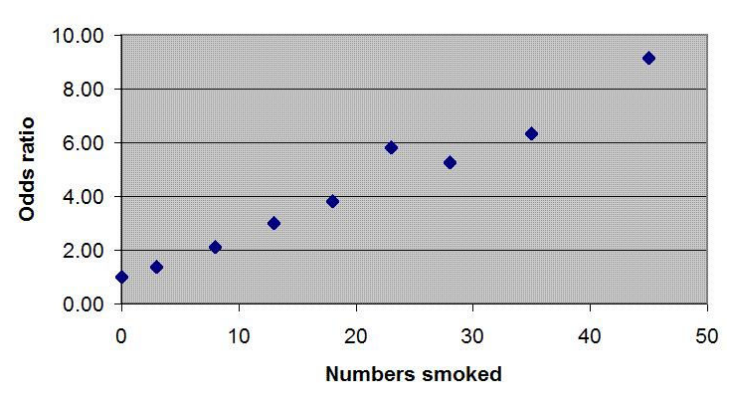
showing the relationship between the odds ratio for first MI and cigarette consumption with respect to non-smoking.

The relationship between the odds ratio for first MI and smoking and cigarette consumption with respect to non-smokers appears non-linear in the original INTERHEART paper.[25] However, as can be seen from Figure 3, if it is assumed that the odds ratio for 20 cigarettes a day is an outlier – there may have been some rounding down in the cigarette consumption to the standard packet size of 20 by either the subjects or observers – then this too is a linear relationship. Linear regression gives an equation:

(7) *OR*_*cig *_= 1 + 0.153145 × *N*

where N is the daily cigarette consumption.

To calculate the odds ratio corrected for the population, the interim odds for the population average cigarette consumption needs to be calculated. The population average cigarette consumption is the cigarette consumption for the whole population, not just smokers. This can be calculated:

Av. cigarette consumption = av. consumption for smokers × proportion that are smokers

The LNOR is then:

$$\xi_{Cig}=\ln\left(\frac{IOR_{Cig}}{POR_{Cig}}\right)$$

#### 2.3.3 Systolic blood pressure

The odds ratios given in the INTERHEART study are for self reported hypertension only. We can make an estimate of the odds ratio by systolic blood pressure versus the average systolic in a non-hypertensive if we assume that the odds are proportional to changes in the systolic blood pressure, and if we know the average values for the hypertensive and non-hypertensive groups. This information was not available to us, so an estimate needed to be made from another source. The average systolic in the ASCOT study was 164 mmHg, which was also the value in a study of home monitoring of Danish hypertensives.[27,28] This seems to be a reasonable estimate for the hypertensive group. Estimating the average value in the non-hypertensive group is more difficult, as this is highly dependent on age and gender. However, a value of 130 mmHg was used as this would be a typical value in the 35 to 64 year old age group in the Health Survey for England.[16]

If we assume that the OR for a hypertensive with a systolic of 164 mmHg is 1.91 (table 3), then the gradient of the function relating the odds ratio to the systolic BP can be calculated as:

(8) $$\frac{\left(OR-1\right)}{\left(HS-NHS\right)}=\frac{\left(1.91-1\right)}{\left(164-130\right)}=0.0268\;\mathrm{per}\;\mathrm{mmHg}$$

Then using the intercept -2.4794 derived from the INTERHEART data we can use the equation to determine the odds ratio for systolic blood pressure with reference to the average normal systolic blood pressure of 130 mmHg:

(9) *OR*_*Syst *_= -2.4794 + 0.0268 × *Systolic*

Armed with the gradient of the line and the intercept, we can calculate the odds ratio for any systolic blood pressure with reference to the assumed normal value of 130 mmHg. The individual odds ratio, IndOR_Syst _is calculated using the individual systolic blood pressure, and the population odds ratio PopOR_Syst _is calculated using the average systolic blood pressure for that age. The LNOR *ξ *for systolic blood pressure can then be calculated using equation (2).

#### 2.3.4 Obesity

The INTERHEART study found that waist-hip circumference ratio (WHR) was a better measure of the contribution of obesity to the risk of first myocardial infarction than body mass index (BMI). However, since data is more readily available for BMI than WHR, a conversion function from BMI to WHR was derived. The function used to estimate waist hip ratio from the BMI and age was derived by linear regression using data from the Health Survey for England:

For men:

(10) *WHR *= 0.409665 + 0.000945*Age + 0.017275*BMI

For women:

(11) *WHR *= 0.714408 + 0.001312*Age + 0.001546*BMI

The INTERHEART team published odds ratios for each of the upper four quintiles of WHR compared to the lowest.[29] The odds ratio is not a linear function of the WHR, so a fractional polynomial was fitted to interpolate the data with reference to the mean WHR of the lowest quintile (Tables 4). The odds ratio for the individual (IOR_WHR_) and the population (POR_WHR_) can then be calculated to give the LNOR:

$$\xi_{WHR}=\ln\left(\frac{IOR_{WHR}}{POR_{WHR}}\right)$$

#### 2.3.5 All other risk factors

All the other risk factors are binary. The beta coefficients are taken as the simple log of the odds, and the adjusted proportion is the value for the individual (1 or 0) minus the proportion affected in the population. So for a 60 year old male diabetic:

*ξ*_*DM *_= ln(OR for diabetics)*(individual value - population prevalence)

*ξ*_*DM *_= ln(2.37)*(1 - 0.081) = 0.793

### 2.4 Implementation

The model was implemented first in Matlab and then in Microsoft Excel to ensure freedom for errors.

## 3 Results

Here we will describe a worked example. We will find the 10-year coronary heart disease risk for a 57 year-old non-diabetic male who smokes 30 cigarettes a day with no personal but a positive family history of cardiovascular disease, a systolic blood pressure of 137 mmHg, a total cholesterol (TC) of 6.2 mmol/l, a high density lipoprotein (HDL) of 1.3 mol/l, and a body mass index of 21. He neither drinks regularly nor exercises. He can give no reliable information about his mental health or fruit and vegetable intake.

### 3.1 ApoB/ApoA1 ratio

The ratio is not known in this case, so must be estimated from the TC/HDL ratio using equation (6).

ApoB/A1_I _≈ TC/HDL/4.41 = (6.2/1.3)/4.41 = 1.0856

The population average ApoB/ApoA1 ratio (BAR_P_) can be estimated from values for the age and gender-adjusted estimates of TC and HDL using the coefficients in Additional file 1, thus:

*PopTC *= 23.8522 - 0.234959 * *Age *+ 0.00093059 * *Age*^2 ^- 510.144 * *Age*^-1 ^+ 4193.01 * *Age*^-2 ^= 5.82

PopHDL = 1.4 (constant with age)

PopApoB/A1 ≈ 5.82/1.4/4.41 = 0.9433

The log odds ratio for the individual (OR_I_) is calculated from equations (5) and (2):

$$\xi_{BA}=\ln\left(\frac{\text{-1}\text{.7+5}\text{.23*1}\text{.0856}}{\text{-1}\text{.7+5}\text{.23*0}\text{.9433}}\right)=0.2073$$

### 3.2 Smoking

The average cigarette consumption in the population for this age and gender is calculated using the coefficients fond in the table in Additional file 1. The product of this and the proportion of smokers for any given age is the population average cigarette consumption. In this case:

N_Cig _= 132944 + 53126.53*Age + 31.019*Age^2 ^- 0.043639*Age^3 ^- 238356*Age^1/2 ^+ 121635.8*ln(Age) - 8444.51*Age.ln(Age) = 16.3

The proportion of smokers at this age is:

S = -4.253823986 + 0.059180468 * Age - 0.00031514 * Age^2 ^+ 149.8257235 * Age^-1 ^- 1666.023544 * Age^-2 ^= 0.2113

Therefore the average number of cigarettes smoked by the population is:

N_Cig _= 0.2113 * 16.3 = 3.4

Using equation (7) the odds ratio (PopOR) is thus:

POR_Cig _= 1 + N_Cig _* 0.153145 = 1.5207

The odds ratio (OR) for the individual is calculated in the same way.

IOR_Cig _= 1 + 30 * 0.153145 = 5.59

And the *ξ*_*Smok *_using equation (2) is

$$\xi_{smok}=\ln\left(\frac{5.59}{1.5207}\right)=1.2979$$

### 3.3 Systolic blood pressure

The population average systolic pressure using the coefficients in Additional file 1 is:

PopSys = -23.669 + 2.43064*Age + -0.010641*Age^2 ^+ 3674*Age^-1 ^+ -32482*Age^-2 ^= 129 mmHg

The reference group for the odds ratios is systolics at or below 115 mmHg, otherwise it is calculated from gradient and intercept as determined in section 2.3.3: (equation 9)

$$\xi_{Syst}=\ln\left(\frac{-2.4794+0.0268\times 137}{-2.4794+0.0268\times 135}\right)$$

### 3.4 Waist hip ratio

The WHR is estimated from the age and the BMI using the equation (10):

WHR = 0.409665 + 0.000945*Age + 0.017275*BMI = 0.8310

The population average for WHR (PopWHR) for a man of his age is:

PopWHR = 0.7193 + 0.0071729*Age - 0.0000536*Age^2 ^= 0.9544

The odds ratio for this individual for the calculated WHR using the coefficients in the Additional file 1 is:

IndOR_WHR _= -41246.21 + 29780.1318*WHR - 8043.6837*WHR^2 ^+ 25335.086*(1/WHR) - 823.58436*(1/WHR^2^) = 0.8310

As this is less than 1.0, the individual WHR is taken as 1.

And for the population average WHR is:

PopOR_WHR _= 1.3207 using the same formula.

The LNOR *ξ *for WHR using equation (2) is thus:

$$\xi_{\text{WHR}}=\ln(\frac{{\text{IndOR}}_{\text{WHR}}}{{\text{PopOR}}_{\text{WHR}}})=\ln(\frac{1}{1.3207})=-0.2781$$

### 3.5 Diabetes mellitus

The baseline probability of having diabetes at this age (PopDM) using the coefficients in the Additional file 1 is:

PopDM = -2.80714 + 0.047436*Age + -0.00025594*Age^2 ^+ 67.419*Age^-1 ^+ -562.65*Age^-2^

PopDM = 0.07476

And the LNOR *ξ *using equation (1) is:

*ξ*_*DM *_= ln(2.37)*(0-0.07476) = -0.0645

### 3.6 Regular alcohol consumption

The baseline probability of being a regular alcohol drinker at this age (P_Alc_) using the coefficients in the Additional file 1 is:

P_Alc _= -3.82669 + 0.076117*Age + -0.000462694*Age^2 ^+ 100*Age^-1 ^+ -889.34*Age^-2 ^= 0.4323

And the LNOR *ξ*_*Alc *_using equation (1) is:

*ξ*_*Alc *_= ln(0.91).(0 - 0.4323) = -0.0535

### 3.7 Psychosocial stress and fruit and vegetable consumption

There is no information available for either of these risk factors, and so an assumption is made that the individual is exactly average for the population. As that means the difference between the individual risk factor value and the mean population value is zero, the LNOR will be zero.

### 3.8 Exercise

The population proportion of exercisers at this age (P_Ex_) using the coefficients in the Additional file 1 is:

P_Ex _= 2.3532 + -0.0205299*Age + 3.23113.10^-6^.*Age^2 ^- 60.944*Age^-1 ^+ 653.57.*Age^-2^

P_Ex _= 0.32542

And the LNOR *ξ*_*Ex *_using equation (1) is:

*ξ*_*Ex *_= ln(0.86).(0 - 0.32542) = 0.0491

### 3.9 Family history of CVD

The baseline probability of having a family history of CVD at this age (P_FH_) is calculated from the prevalence of CVD in men at age 57 (CVD_42_):

FH_CVD _= 1 - (1-CVD_42_)^2 ^= 0.24

And the LNOR *ξ*_*FH *_using equation (1) is:

*ξ*_*FH *_= ln(1.45)*(0-0.24) = -0.0892

#### Calculating mortality

The 10 year mortality rate BM_10 _can be calculated as:

$$1-(e^{\left(-\sum_{i=Age}^{i=Age+10}BM_{i}\right)})$$

The baseline mortality odds (BMO) is therefore:

(12) $$BMO=\frac{1-e^{-\sum_{i=Age}^{i=Age+10}BM_{i}}}{-e^{-\sum_{i=Age}^{i=Age+10}BM_{i}}}$$

and the baseline mortality at a given age *i *(BM_*i*_) is found using the set of Normal distribution curves with the means and standard deviations in set A:

A = {(20 5), (30-5), (40-5), (50-5), (60-5), (70-5), (80-5), (90-5), (25-10), (45-10), (65-10), (85-10), (20 15), (50 15), (80 15), (90 30)}

And the following set of coefficients C from the Table 2, the first of which is a constant:

C = {0.028432143 0.02783794 0.021060258 0.020598088 0.019311202 0.011118031 0.021130765 0.015343166 -0.037214697 0.223830756 0.193671366 0.026298386 0.160344847 -1.384157929 -1.857484078 -2.899112366 5.065048492}

BM_10 _= 0.0024886

The prevalence of CHD is calculated in a similar manner using the coefficients in the Table 2 to give:

BM_0 _= 0.00493/(1-0.00493) = 0.0025

Pr_CHD _= 0.01483

We can then correct the mortality for the presence or absence of CVD, using equation:

$${\hat{BM}}_{i}=\frac{0.0025}{\left(1-0.01483\right)+3.3\times 0.01483}=0.00107$$

Converting this probability to odds, the value remains at 0.0048.

We then calculate the λ as the sum of all the LNORs using equation (3):

λ = 1.2979 - 0.0645 + 0.0463 + 0.2073 + 0.0491 - 0.2781 + 0.2824 + 0.0514 = 0.2973

Our adjusted odds for death are thus:

Odds for death in 10 years from CHD = e^λ*0.2973 ^= 0.0254

This is then converted back from odds to probabilities:

The 10-year probability of death from CHD = 0.0254/(1+0.0254) = 0.0247 = 2.47%

## 4 Discussion

This exercise demonstrates how published information can be used to construct a mathematical model of cardiovascular risk. The same methods should be applicable to other disease groups where there is sufficient information available. The method requires: the odds ratios for each of the risk factors when controlled for all other risk factors; mortality rates and prevalences for the diseases of interest; and prevalence rates and mean values for the risk factors in the relevant population.

Before use, this model must be tested in different populations to assess its accuracy. The results of the INTERHEART study would suggest that it should be applicable in different geographical locations and to different ethnic groups without adjustment, since the predictions are anchored in a dataset for which there is a great deal of information on mortality rates and mean values. The INTERHEART study would suggest the residual variation at a population level is significantly less than ten percent. However, it should still be possible to apply the same principles by substituting the mortality data and the prevalence data for any population where that information is available to improve accuracy.

A major advantage of this model is the comprehensive set of independent risk factors used. It is likely that other risk factors have very little residual independence once all these factors are taken into account. For example, social and economic deprivation is included in other CVD and CHD models such as QRisk and Assign.[9,10] The INTERHEART study was conducted in 52 countries including low and middle income countries, and yet this factor did not emerge as significant when all nine risk factors were included. Equally, country and ethnicity did not remain as independent risk factors suggesting that the odds ratios derived are applicable in all 52 countries. It would seem plausible that the odds ratios are also applicable in countries not included in this study.

### 4.1 Limitations

#### 4.1.1 Assumptions

A large number of assumptions were made in the construction of this model. The more important assumptions that might limit the accuracy of the model are described below.

##### 4.1.1.1 That the underlying pathological processes and aetiological factors are the same for atheromatous disease, whether it is myocardial infarction, cerebrovascular or angina pectoris. This excludes death from haemorrhagic stroke

The odds ratios for the different cardiovascular pathologies should be highly correlated because there is a common underlying process at work, the formation of atheroma. However, there may be variations that are specific to certain pathologies, such as atrial fibrillation and stroke. In the INTERHEART study the subjects had experienced a first MI. Factors such as blood viscosity have a greater impact on MI than chronic ischaemia. It is possible that some of the modeled risk factors – such as psychosocial stress – may affect MI and chronic ischaemia, in different ways.

We assume that the risk factor profiles and odds ratios for those risk factors are similar in those who die from an MI before reaching hospital and those who survive. In a cross-sectional study like INTERHEART, the outomes are not entirely equivalent to the prospective predictions of death from MI or CHD. In the INTERHEART study, subjects were identified on presentation with a first MI. Many potential subjects will have not survived to be recruited into the study. If there are significant differences between those that survive to hospital and those that don't, then some error will be generated in this model.

##### 4.1.1.2 That the odds ratios for each risk factor are the same for those with and without CVD

We assume that the pathological processes affecting progression of asymptomatic, mild atheroma to symptomatic, moderate atheroma, are essentially the same as those causing further progression of existing moderate atheroma, and that the scale of effect of the different risk factors is the same at all stages of disease. This is not necessarily true, different risk factors might have particular significance at difference stages of disease. For example it is possible that some risk factors have a particular role in plaque rupture, or thrombus formation, and less in plaque formation.

##### 4.1.1.3 That the odds ratios apply equally to all populations regardless of geography, ethnicity or socioeconomic group

The INTERHEART study found that, once all nine final risk factors were included, country, and ethnicity and socioeconomic group did not have a significant effect. The well recognized ethnic, geographic and socioeconomic differences on CVD must therefore be mediated by the included risk factors. To apply the odds ratios to a given population, what we require, is the mortality rate in that population and the average value for all the included risk factors in that population at that time. We have used the mortality of CHD in the UK in 2003, and the average values for the risk factors used, in the UK in 2003. These will differ from the mortality and average risk factor values in the INTERHEART study. However, if this result of the INTERHEART study, that the odds ratios apply regardless of geography and ethnicity, is robust then this should not affect the results.

##### 4.1.1.4 That hypertensives had an average systolic of 164 mmHg and non-hypertensives had an average systolic of 130 mmg in the INTERHEART study and the relationship between the odds ratio and systolic blood pressure is linear

With regard to the systolic values for hypertensives and non-hypertensives, this is very uncertain whether this assumption is accurate, but is based on data from two different countries, and would seem to be reasonable assumptions in the absence of better information. Data on the precise values in the INTERHEART study would possibly improve the model and increase confidence in it.

The systolic blood pressure was modeled here with an assumption of a linear relationship with the odds ratio. However, the results in Lewington et al 2002 would suggest that the age-adjusted absolute risk varies on a doubling scale with systolic blood pressure [30] and, previous work would suggest that this linear relationship does not hold for very severe hypertensives.[31] The INTERHEART study was unable to determine the relationship between the systolic and odds ratio adjusted for all nine risk factors and so we felt an assumption of a linear relationship was reasonable.

##### 4.1.1.5 That the relative risk for coronary heart disease death in those with pre-existing CVD is 3.3, regardless of the type of pre-existing CVD

This is a weak assumption, and based on a figure after Kannel.[21] Different types of existing cardiovascular disease will have different degrees of impact on risk.[31] The figure found by Kannel may be an average of these differing values. The value of this assumption will need to be tested in an evaluation of the model on external data.

##### 4.1.1.6 That the odds ratios for the different risk factors remain constant over time and at different ages

The odds ratios given in the INTERHEART study relate to the occurrence of first MI, and the risk factor data was collected at that time. It is unclear how those odds ratios differ over time and with the age of subjects. Also, older subjects will have higher risks of competing causes of death, and this may in turn affect the odds ratios for the risk factors predicting CVD.

##### 4.1.1.7 Other limitations

Using a wider range of risk factors can reduce the accuracy of the model if the available data on the additional risk factors is poor. Models developed using fewer risk factors embed information pertinent to the missing risk factors within the regression coefficients for factors that interact with the missing risk factors. With the larger models, the coefficients will have been regressed in the presence of those risk factors and so if that information is missing – for example if patients' fruit and vegetable consumption is not recorded in a dataset – their effect is lost to the model.

Body mass index is used an approximation to waist-hip ratio, and TC/HDL ratio as a proxy for ApoB/ApoA1 ratio. Use of these proxy measures will reduce the accuracy of the model and waist-hip ratio and the ApoB/A1 ratio should be used in preference, when available.

Individuals at high risk of CHD are often at high risk from competing causes of death. Consequently, some individuals at high risk may die from another cause prior to a predicted CHD event. This could lead to overestimation of risk from CHD in those at highest risk.

Our method takes a population mortality rate and prevalence rates for CVD and adjusts them using the mean values for risk factors in the given population. This is valid provided the distribution of risk factor values is not heavily skewed and the relationship between the risk factor values and mortality rates obey the assumptions described above.

## 5 Conclusion

This paper demonstrates how a comprehensive, mixed odds model can be constructed using widely available information and without access to training data sets. The method could be useful in modeling a broad range of disease areas. Further research needs to be done to evaluate the accuracy of the model in different population groups using historical cohort data.

## 6 Competing interests

Dr Martin is the author and owner of the intellectual property rights of the Laindon Survival Model. He also works for RMS Ltd, a risk modeling company.

## 7 Authors' contributions

CJM conceived of and designed the model, and is the principal author of the paper. PT supervises CJM's PhD and contributed to the paper. HWWP gave statistical advice and contributed to the paper.

## Pre-publication history

The pre-publication history for this paper can be accessed here:



## Supplementary Material

Additional file 1 **Tables showing the coefficients for the fractional polynomials.** These tables give the values of the coefficients for men and for women, for each function making up the fractional polynomial. Each fractional polynomial describes the value of a risk factor at a given age. Click here for file
