# Supplementary material for: Construction of an odds model of coronary heart disease using published information: the Cardiovascular Health Improvement Model (CHIME)
Source: BMC Med Inform Decis Mak. 2008 Oct 31;8:49. doi: 10.1186/1472-6947-8-49 (PMC2601038; doi:10.1186/1472-6947-8-49)
Supplement: Additional file 1 — Tables showing the coefficients for the fractional polynomials. These tables give the values of the coefficients for men and for women, for each function making up the fractional polynomial. Each fractional polynomial describes the value of a risk factor at a given age. [file 1472-6947-8-49-S1.docx]

| **Function of age (x)**  **Risk factor** | **Men** | | | | | | | | |
| --- | --- | --- | --- | --- | --- | --- | --- | --- | --- |
|  | **Constant** | **x** | **x^2^** | **x^3^** | **$\sqrt{x}$** | **x^-1^** | **x^-2^** | **x.ln(x)** | **ln(x)** |
| **Smoking** | -4.253823 | 0.0591804 | -0.00031514 | 0 | 0 | 149.8257 | -1666.0235 | 0 | 0 |
| **Number of cigarettes** | 132944 | 53126.53 | 31.01909 | -0.04363924 | -238356 | 0 | 0 | -8444.511 | 121635.8 |
| **Diabetes** | -2.8071383 | 0.0474358 | -0.0002559 | 0 | 0 | 67.418949 | -562.64992 | 0 | 0 |
| **Daily fruit & veg’ consumption (%)** | -2.0669267 | 0.0486666 | -0.000269 | 0 | 0 | 72.504979 | -645.59111 | 0 | 0 |
| **Exercise regularly (% >3.5 hours per week)** | 2.3531616 | -0.0205298 | 3.2311*10^-6^ | 0 | 0 | -60.944018 | 653.56012 | 0 | 0 |
| **Total cholesterol (TC)** | 23.852215 | -0.2349594 | 0.00093059 | 0 | 0 | -510.1446 | 4193.0172 | 0 | 0 |
| **High Density Lipoprotein (HDL)** | 1.4 | 0 | 0 | 0 | 0 | 0 | 0 | 0 | 0 |
| **Hip circumference (cm)** | 6.158358 | 1.783149 | -0.010924 | 0 | 0 | 2003.9018 | -21025.056 | 0 | 0 |
| **Population average waist hip ratio** | 0.7193 | 0.007179 | - 0.0000536 | 0 | 0 | 0 | 0 | 0 | 0 |
| **WHR odds** | -41246.21013 | 29780.13175 | -8043.683686 | 0 | 0 | 25335.08639 | -5823.584363 | 0 | 0 |
| **Height (m)** | 176.652597 | 0.0371594 | -0.0016242 | 0 | 0 | 30.624557 | -428.8653 | 0 | 0 |
| **Weight (Kg)** | 47.001716 | 1.275054 | -0.0120946 | 0 | 0 | -92.570514 | 595.83393 | 0 | 0 |
| **Systolic blood pressure (mm Hg)** | -23.669404 | 2.4306416 | -0.0106406 | 0 | 0 | 3674.8611 | -32482.851 | 0 | 0 |
| **Drinks alcohol regularly (%)** | -3.8266892 | 0.0761169 | -0.0004626 | 0 | 0 | 100.06154 | -889.34187 | 0 | 0 |
| **Psychosocial stress (% high GHQ score)** | 5.8260412 | -0.0943892 | 0.00053894 | 0 | 0 | -140.64973 | 1191.67306 | 0 | 0 |

| **Function of age (x)**  **Risk factor** | **Women** | | | | | | | | |
| --- | --- | --- | --- | --- | --- | --- | --- | --- | --- |
|  | **Constant** | **x** | **x^2^** | **x^3^** | **$\sqrt{x}$** | **x^-1^** | **x^-2^** | **x.ln(x)** | **ln(x)** |
| **Smoking** | 2.63886961 | -0.0308870 | 0.00010191 | 0 | 0 | -66.438921 | 628.129755 | 0 | 0 |
| **Number of cigarettes** | -26313.9 | -211539 | -2179.8 | 53.8925 | 219448.7 | 0 | 0 | 99119.83 | -78579.5 |
| **Diabetes** | -2.8071384 | 0.04743586 | -0.000256 | 0 | 0 | 67.4189492 | -562.64993 | 0 | 0 |
| **Daily fruit & veg’ consumption (%)** | -1.7680263 | 0.04426946 | -0.00025 | 0 | 0 | 66.2013807 | -586.95687 | 0 | 0 |
| **Exercise regularly (% >3.5 hours per week)** | 2.64127901 | -0.0248236 | 0.00002675 | 0 | 0 | -73.004079 | 718.073385 | 0 | 0 |
| **Total cholesterol (TC)** | 16.2617788 | -0.0825169 | -0.00001315 | 0 | 0 | -393.410737 | 3867.424038 | 0 | 0 |
| **High Density Lipoprotein (HDL)** | -4.3913653 | 0.109454908 | -0.00066914 | 0 | 0 | 13.46393694 | -1064.63879 | 0 | 0 |
| **Hip circumference (cm)** | -132.98699 | 3.685093118 | -0.02028498 | 0 | 0 | 5311.239251 | -47795.4294 | 0 | 0 |
| **Population average waist hip ratio** | 0.74428571 | 0.00175 | -0.00000357 | 0 | 0 | 0 | 0 | 0 | 0 |
| **WHR odds** | -4897.856982 | 3625.051788 | -992.8801578 | 0 | 0 | 2911.017547 | -642.8578511 |  |  |
| **Height (m)** | 138.0645186 | 0.554316816 | -0.0051231 | 0 | 0 | 511.7362653 | -3597.22577 | 0 | 0 |
| **Weight (Kg)** | -113.900425 | 3.327277772 | -0.02110928 | 0 | 0 | 4402.288956 | -39811.8214 | 0 | 0 |
| **Systolic blood pressure (mm Hg)** | -195.468854 | 5.114034915 | -0.02309628 | 0 | 0 | 7289.384095 | -59456.6994 | 0 | 0 |
| **Drinks alcohol regularly (%)** | 3.048434629 | -0.03276474 | 0.000112488 | 0 | 0 | -82.5709932 | 760.2298873 | 0 | 0 |
| **Psychosocial stress (% high GHQ score)** | 5.82604126 | -0.0943892 | 0.00053893 | 0 | 0 | -140.64973 | 1191.67306 | 0 | 0 |
